# Aflibercept Suppression of Angiopoietin-2 in a Rabbit Retinal Vascular Hyperpermeability Model

**DOI:** 10.1167/tvst.12.5.17

**Published:** 2023-05-16

**Authors:** Claudia Lange, Reimo Tetzner, Tobias Strunz, Kay D. Rittenhouse

**Affiliations:** 1 Research & Development, Precision Medicine Markers, Bayer AG, Berlin, Germany; 2 Research & Development, Biosample Operation Management and Assay Technologies, Bayer AG, Berlin, Germany; 3 Research & Development, Biomedical Data Science II, Bayer AG, Wuppertal, Germany; 4 Medical Affairs, Bayer Consumer Care AG, Basel, Switzerland

**Keywords:** mechanism-of-action, anti-VEGF, ANG2, retinal disorders, hyperpermeability model

## Abstract

**Purpose:**

Anti-vascular endothelial growth factor (anti-VEGF) therapies, which attenuate the capacity of VEGF to bind to VEGF receptors, are standard-of-care options for various retinal disorders that are characterized by pathologic retinal angiogenesis and vascular permeability. Multiple receptors and ligands have also been reported as being involved in these pathways, including angiopoietin-1 (ANG1) and angiopoietin-2 (ANG2).

**Methods:**

Electrochemiluminescence immunoassays were used to detect human VEGF (hVEGF), as well as rabbit ANG2 and basic fibroblast growth factor protein levels in vitreous samples derived from a study evaluating the efficacy of the anti-VEGF agents ranibizumab, aflibercept, and brolucizumab in an hVEGF165-induced rabbit retinal vascular hyperpermeability model.

**Results:**

hVEGF was completely suppressed in rabbit vitreous after anti-VEGF treatment for 28 days. ANG2 protein in vitreous and *ANGPT2* mRNA in retina tissue were similarly suppressed, although the anti-VEGF agents do not directly bind to ANG2. Aflibercept demonstrated the greatest inhibitory effect in ANG2 levels in vitreous, which correlated with strong, durable suppression of intraocular hVEGF levels.

**Conclusions:**

This study explored the effects of anti-VEGF therapies beyond direct binding of VEGF by evaluating protein levels and the expression of target genes involved in angiogenesis and associated molecular mechanisms in the rabbit retina and choroid.

**Translational Relevance:**

In vivo data suggest that anti-VEGF agents currently used for the treatment of retinal diseases could provide beneficial effects beyond direct binding of VEGF, including suppression of ANG2 protein and *ANGPT2* mRNA.

## Introduction

The pathophysiology of retinal disorders, such as neovascular age-related macular degeneration (nAMD), diabetic macular edema (DME), and macular edema secondary to retinal vein occlusion (RVO), is complex and likely involves a combination of genetic, metabolic, and environmental components.^1^^–^^3^ The retina has a high metabolic demand,^4^ and hypoxic conditions incite local inflammation.^5^^,^^6^ In nAMD, a number of proteins, including vascular endothelial growth factor (VEGF, specifically VEGF-A), at the retinal pigment epithelium and Bruch's membrane become dysregulated and cause neovascularization of choroidal vessels.^7^ Pathological blood vessels formed in the retina by neovascularization due to hypoxia-induced progression of retinal vascular diseases, such as diabetic retinopathy/DME and RVO, are immature and leaky.^5^ The pathologic hypoxic microenvironment that abnormal vessel growth creates is associated with the overexpression of multiple factors.^5^^,^^8^ These include placental growth factor (PlGF), which is a ligand that solely binds VEGF receptor 1 (VEGFR-1) and can uniquely mediate inflammatory cascades that may remain active beyond VEGF activity,^9^ and VEGF, ligands that are involved in signaling via the VEGF receptors VEGFR-1 and VEGFR-2.^8^ Targeting this dysregulated VEGF signaling (and thereby also targeting angiogenesis, vascular permeability, and edema) has been the focus of the development of novel treatments for retinal disease.

Anti-VEGF therapies have demonstrated clinically meaningful improvements for patients with retinal (and choroidal) disorders.^10^^–^^14^ Aflibercept is a recombinant fusion protein, consisting of the extracellular domains of human VEGFR-1, which specifically and tightly binds to VEGF, VEGF-B, and PlGF, and human VEGFR-2, which specifically and tightly binds to the key VEGF ligand.^15^ These receptor extracellular domains are fused to the fragment crystallizable (Fc) portion of human immunoglobulin G1 (IgG1).^15^ Aflibercept binds VEGF, VEGF-B, and PlGF, sequestering these molecules and thus reducing signaling via VEGF receptors.^15^ Ranibizumab is a humanized monoclonal IgG1κ isotype fragment (Fab; lacking an Fc region), which binds VEGF,^16^ and brolucizumab is a humanized monoclonal single-chain variable domain antibody fragment, which also binds VEGF.^17^

Although the VEGFR pathway is described as being central to the progression of retinal disorders, the TIE2 receptor pathway (the target for angiopoietin-2 [ANG2] and angiopoietin-1 [ANG1]) constitutes another component of the vascular development system.^1^ Similar to VEGF, ANG2 is upregulated by hypoxia^18^^,^^19^ and has been reported to have a role in destabilizing vessels through competition with ANG1 and inhibition of the ANG1 receptor TIE2 in preclinical studies evaluating retinal and choroidal vascular diseases.^19^^–^^21^ In a process described as an angiogenic switch, the presence or absence of VEGF determines the outcome of ANG2 stimulation—when VEGF levels are elevated, ANG2 levels are also elevated.^22^ ANG2 binds to TIE2, blocking TIE2 downstream signaling and leading to vessel remodeling, exacerbation of VEGF-induced pathologic angiogenesis, and vascular permeability.^22^ In the absence of VEGF, ANG2 promotes endothelial cell death and vessel regression^21^^,^^22^; thus a specific environmental influence on its activity has been described. Furthermore, elevated levels of ANG2 and VEGF have been observed in eyes with retinal disorders,^23^^–^^25^ and multiple studies have demonstrated that both are co-regulated in retinal angiogenesis.^23^^–^^25^ The potential role of ANG2 in retinal neovascularization has led to an interest in this pathway in drug development for retinal vascular disease.

Beyond its anti-VEGF activity, aflibercept has been shown clinically and experimentally to modulate additional factors, such as PlGF,^26^ galectin-1,^27^ and ANG2, and effects such as retinal inflammation in an oxygen-induced retinopathy model in mice.^2^ Given the central role of VEGF in retinopathy and the ANG2-mediated context-dependent antagonism of TIE2 (exacerbating vascular disease), targeting both ligands could potentially provide additional benefits in vascular retinal disease.^28^

Clinical trials for nesvacumab, a fully human IgG monoclonal anti-ANG2 antibody (with the highest reported binding affinity to the ANG2 ligand^29^) co-administered with aflibercept, failed to demonstrate superiority of gains in visual acuity in both nAMD (ONYX^30^) and DME (RUBY^31^), and further development was halted. Faricimab, which was first approved by the U.S. Food and Drug Administration (FDA) in early 2022,^32^ is a bispecific monoclonal antibody that binds VEGF (via its ranibizumab arm) and ANG2.^23^^,^^33^ Recent clinical trials have shown non-inferiority in mean visual acuity gains when compared with aflibercept in patients with nAMD with varying treatment intervals.^34^ Understanding the actions of aflibercept and other anti-VEGF agents in a retinopathy model would help to further elucidate whether modulation of ANG2 and other additional factors directly or indirectly impacts the development or progression of retinopathy.

Schubert et al.^35^ investigated the relationships among the molecular properties, ocular pharmacokinetics, and pharmacology of the anti-VEGF agents aflibercept, brolucizumab, and ranibizumab to characterize their efficacy in a preclinical rabbit model of retinopathy. They also mathematically modeled the efficacy of the agents across their in vivo and in vitro study results. Using their integrated, in silico/in vitro/in vivo approach, the rank orders of efficacy of the three anti-VEGF agents ranged from aflibercept as most effective in preventing retinal microvascular leakage to ranibizumab as least effective, suggesting differences in efficacy that are contingent on molecular, pharmacokinetic, potency, and posology differences. Similarly, the anti-angiogenic activity of these anti-VEGF agents may also be related to their biochemical characteristics.

This study used ocular samples generated from the human VEGF-165 (hVEGF)-induced rabbit retinal vascular hyperpermeability model from Schubert et al.^35^ to elucidate the effects of anti-VEGF treatment on free hVEGF levels in vitreous. Ocular samples were obtained from rabbit cohorts completing their terminal cycles of hVEGF-induced leakage. The time points available for evaluation were days 28 and 56; vascular leakage suppression activity was lost by day 56 for all anti-VEGF agents.^35^ The effects of anti-VEGF treatment were also evaluated by quantification of vitreous protein levels and gene expression profiling of selected target genes involved in angiogenesis, inflammation, and vascular permeability and edema.

## Methods

### Rabbit Retinal Vascular Hyperpermeability Model

In brief, following quarantine and acclimation, Dutch belted rabbits (Iris Pharma, La Gaude, France) were randomized into study groups containing seven rabbits per time point. Masked clinical doses of ranibizumab (0.25 mg; Genentech, San Francisco, CA), aflibercept (1 mg; Regeneron, Tarrytown, NY), and brolucizumab (3 mg; Novartis Pharma, Basel, Switzerland) were administered (as a 25 µL injection, resulting in 50% of the clinical dose) at baseline in the vitreous fluid of the rabbit's eye. Induction of leakage was produced via 50 µL intravitreal injection of 500 ng recombinant human VEGF (rhVEGF-165) at day 5 (1 week) and day 26 (4 weeks) in group 1, and at day 33 (5 weeks) and day 54 (8 weeks) in group 2. Schubert et al.^35^ described a further group that received VEGF injections at day 40 (6 weeks) and day 68 (10 weeks). At 47 hours (±3 hours) after induction, retinal fluorescein angiography was performed; after the second fluorescein angiography cycle (days 28, 56, and 70), animals were euthanized and vitreous, retina, and choroid samples were collected. As suppression of leakage was lost (according to Schubert et al.^35^) at day 56, ocular tissue samples from day 70 were excluded from further analysis. Reference samples (untreated reference) were obtained from Dutch belted rabbits commercially obtained from BioIVT (Burgess Hill, UK). Samples were assessed to determine if protein and gene expression of relevant target genes was modulated under conditions of hVEGF-induced retinal vascular leakage and whether each anti-VEGF agent (none of which directly binds to ANG2) affects any components of the ANG2/TIE2 pathway and other relevant targets associated with VEGF, TIE2, angiogenesis, inflammation, vascular permeability, and edema.

### Determination of Protein Concentration

Electrochemiluminescence immunoassays (ECLIAs) were used to detect hVEGF, rabbit fibroblast growth factor 2 (FGF2), and rabbit ANG2 protein levels in vitreous samples derived from rabbits (Meso Scale Diagnostics, Rockville, MD). The detection of VEGF and FGF2 was performed as part of the multiplex Angiogenesis Panel 1 Kit K15190D (Meso Scale Diagnostics). The frozen vitreous humor was thawed and prediluted 1:100; 50 µL prediluted sample was added per well in duplicate. The detection of free, unbound fraction of VEGF after anti-VEGF treatment was confirmed by additional drug-interaction titration experiments. Thus, only free hVEGF was detected using this assay. No cross-reactivity with rabbit recombinant VEGF was observed (Abcam, Cambridge, UK). ANG2 levels were determined using the human ANG2 ECLIA K151YRR-2 (Meso Scale Diagnostics) with a 25 µL sample input after 1:2 dilution. Rabbit recombinant ANG2 protein (Abcam, Cambridge, UK) was used for generating a standard/reference curve. Human and rabbit FGF2 and ANG2 proteins share 99% and 91% homology, respectively.

### Determination and Analysis of mRNA Expression

RNA was isolated from retina and choroid tissue of rabbits after hVEGF challenge and/or anti-VEGF treatments using a Maxwell RSC simplyRNA Tissue Kit on a Maxwell RSC48 instrument (Promega, Madison, WI). RNA was quantified using the QuBit RNA HS Assay Kit (Thermo Fisher Scientific, Waltham, MA). Target gene expression was analyzed using NanoString technology, which allows the direct tagging (molecular barcoding technology) and counting of mRNA molecules (NanoString Technologies, Amsterdam, The Netherlands). A custom-made code set containing 35 target genes involved in angiogenesis and associated molecular mechanisms was designed and manufactured by NanoString. The code set additionally contained five housekeeping genes to correct for RNA input quantity and quality differences. Reporter and Capture ProbeSets were incubated together with 200 ng RNA in hybridization buffer. The samples were then loaded onto the NanoString GEN2 Prep Station according to the manufacturer's instructions and scanned on the NanoString nCounter Digital Analyzer. The raw data were processed by using the nSolver software for internal quality control processes and were normalized using the housekeeping genes according to the manufacturer's specifications. Principal component analysis (PCA) was performed to detect potential outlier samples at a threshold of being away more than two standard deviations of a group's mean. One outlier sample was identified in the untreated choroid reference group when compared with all other untreated reference samples. This outlier was excluded from further investigations (Supplementary Fig. S1, top left, and Supplementary Table S1).

### Statistics

Dunnett's multiple comparisons test (one-sided) was performed to assess whether there were significant differences between the vehicle-treated and the anti-VEGF–treated groups. Normal distribution of data and similar variance were assumed, given equivalent sample sizes per group. Due to the small sample size the data was not formally tested for normality. Before assuming normality data was visualized (e.g. Q-Q-plot) to support the claim. Follow-up analyses of the mRNA data were performed using the NanoTube library (version 1.2.0) with default settings in R 4.2.1 (R Foundation for Statistical Computing, Vienna, Austria). Comparison to negative controls was disallowed as samples from multiple tissues were included in the analysis. Statistical testing was performed using an empirical Bayesian method, and correction for multiple testing was applied according to Benjamini and Hochberg.^36^ PCA was performed based on the function NanoStringPCA (NanoTube library). All gene symbols shown correspond to the National Center for Biotechnology Information RefSeq library database.^37^

### Study Approval

Schubert et al.^35^ examined the molecular versus in vivo properties of aflibercept, brolucizumab, and ranibizumab using a Dutch belted rabbit model of retinal vascular hyperpermeability. All in vivo rabbit experiments were conducted according to Directive 2010/63/UE of the European Convention for the Protection of Vertebrate Animals Used for Experimental and Other Scientific Purposes, and the ARVO Statement for the Use of Animals in Ophthalmic and Vision Research.

## Results

### Significant Suppression of Free hVEGF Levels in Rabbit Vitreous Following Anti-VEGF Administration

**Figure 1.**
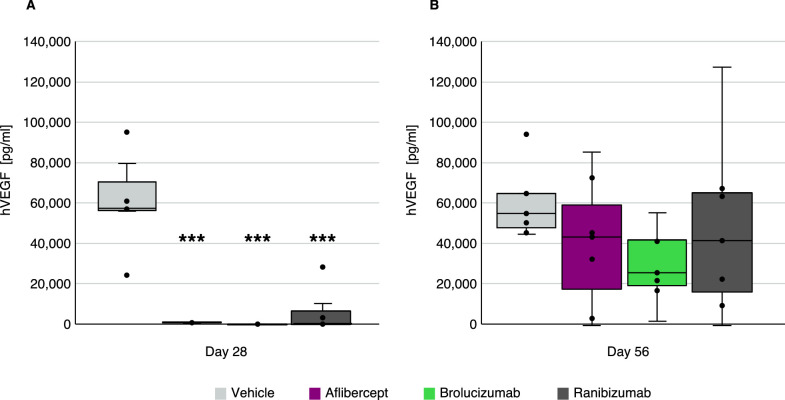
Significant suppression of free hVEGF levels in hVEGF-challenged rabbit vitreous 28 days (A) and 56 days (B) after anti-VEGF treatment. hVEGF levels (pg/mL) on day 28 (A) and on day 56 (B) are shown after treatment with vehicle or anti-VEGF agents (*n* = 7 per group). ***P < 0.001.

ECLIA analysis demonstrated that hVEGF vitreous levels were elevated in the vehicle control group following intravitreal administration of hVEGF to the rabbits. hVEGF levels were almost 10,000-fold above vitreous levels quantified in the anti-VEGF-treated groups (Fig. 1). At day 28 (i.e., 28 days after anti-VEGF treatment and 2 days after hVEGF challenge at day 26), a large suppression of free, unbound hVEGF levels was detected in the rabbit vitreous (Fig. 1A). This effect was observed with all three anti-VEGF treatments at this time point. At day 56, the anti-VEGF therapies showed numerically lower levels of VEGF compared to the vehicle group, but with high intra-treatment group variability (Fig. 1B).

### Suppression of ANG2 Protein and *ANGPT2* mRNA After Anti-VEGF Treatment

**Figure 2.**
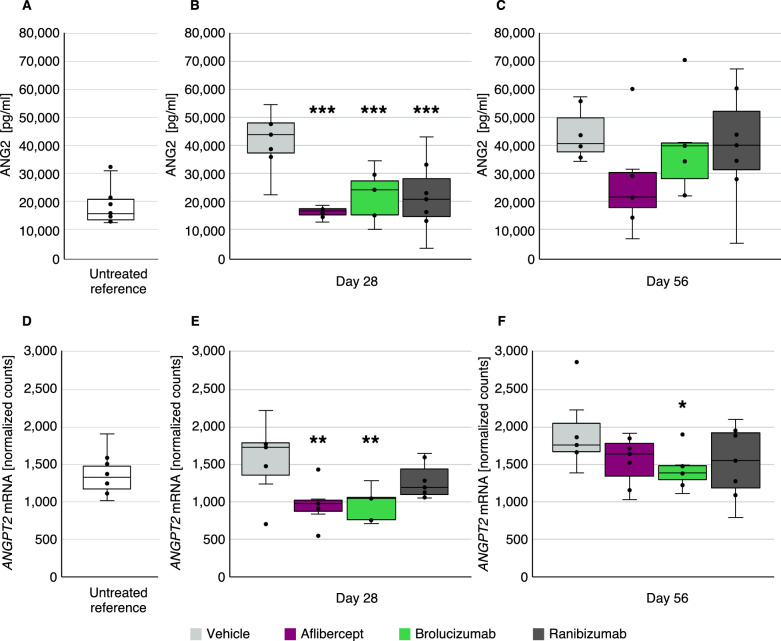
Anti-VEGF treatment suppresses ANG2 elevation in vitreous (A–C) and retina (D–F) caused by intravitreal hVEGF challenge. ANG2 protein levels (pg/mL) were detected by ECLIA in vitreous samples of untreated reference rabbits (*n* = 10) (A) and after hVEGF challenge in vehicle or anti-VEGF treatment on day 28 (*n* = 7) (B) and on day 56 (*n* = 7) (C). *ANGPT2* gene expression was determined by mRNA analysis (normalized counts) in retina (D) tissues derived from untreated reference rabbits (*n* = 10) and after VEGF challenge and vehicle or anti-VEGF treatment on day 28 (*n* = 7) (E) and day 56 (*n* = 7) (F) in the retina. *P < 0.05; **P < 0.01; ***P < 0.001.

ANG2 protein and *ANGPT2* mRNA levels were elevated following challenge with hVEGF in vitreous from vehicle-treated rabbits (Figs. 2B, 2C, 2E, 2F) compared to untreated reference Dutch belted rabbits in the ECLIA analysis (Fig. 2A) and NanoString analysis (Fig. 2D), respectively. The median vitreous protein concentration of ANG2 in the untreated control rabbits was ∼15,000 pg/mL, consistent with previously published literature (Wong et al. *IOVS*. 2018;59:ARVO E-abstract 5261).^38^ This was approximately threefold lower than the levels in hVEGF-challenged rabbit vitreous (∼45,000 pg/mL) (Figs. 2A–2C). Anti-VEGF treatment resulted in significant suppression of elevated ANG2 protein levels (*P* < 0.001) by day 28 (Fig. 2B). In addition, the *ANGPT2* mRNA increase was significantly suppressed by aflibercept and brolucizumab on day 28 in the retina (Fig. 2E), as well as in choroid tissue by all anti-VEGF agents (Supplementary Figs. S2A, S2B). This effect was reduced or lost by day 56 at the protein and mRNA levels in retina (Figs. 2C, 2F) and choroid tissue (Supplementary Fig. S2C).

### Suppression of Basic FGF2 Protein and mRNA After Anti-VEGF Treatment

**Figure 3.**
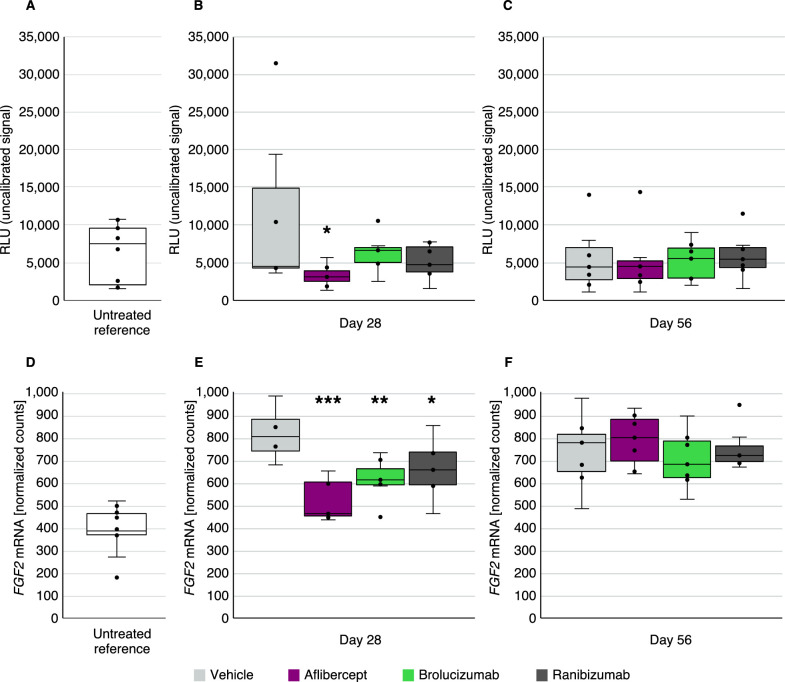
Suppression of FGF2 in hVEGF-challenged rabbit vitreous (protein; A–C) and retina (mRNA; D–F) 28 and 56 days after anti-VEGF treatment. FGF2 protein levels were detected by ECLIA in vitreous samples of untreated reference rabbits (*n* = 10) (A) and after hVEGF challenge and vehicle or anti-VEGF treatment on day 28 (*n* = 7) (B) and on day 56 (*n* = 7) (C) . *FGF2* gene expression was determined by mRNA analysis in retina (D) tissues derived from untreated reference rabbits (*n* = 10), as well as after VEGF challenge and vehicle or anti-VEGF treatment on day 28 (*n* = 7) (E) and day 56 (*n* = 7) (F) in the retina. *P < 0.05; ** *P* < 0.01; *** *P* < 0.001. RLU, relative luminescent units.

Baseline FGF2 protein levels in vitreous and mRNA levels in retina, obtained from untreated reference Dutch belted rabbits, are shown in Figs. 3A and 3D, respectively. ECLIA analysis demonstrated suppression of FGF2 protein levels in the vitreous of aflibercept-treated rabbits (*P* < 0.05) (Fig. 3B). This finding was confirmed by NanoString analyses, which showed significantly lower expression of *FGF2* mRNA following anti-VEGF agent administration in the retina (Fig. 3E) and choroid (Supplementary Fig. S2E) through day 28, with aflibercept showing the most significant effect (retina, *P* < 0.001; choroid, *P* < 0.05) on *FGF2* mRNA in the retina on day 28. The other anti-VEGF agents did not significantly suppress FGF2 protein in vitreous, whereas both showed significant, although less robust, *FGF2* mRNA suppression in the retina. This effect was lost by day 56 (Figs. 3C, 3F, Supplementary Fig. S2F).

### Differential Expression of Genes in Retina and Choroid Tissue Derived From Untreated Reference Rabbits and hVEGF Challenged Rabbits

The differential expression of 35 pre-selected target genes was evaluated in retina compared to choroid tissue, as well as the effect of vascular leakage induction by using hVEGF. Figure 4A depicts PCA showing the distribution of choroid, retina, muscle, and pooled retina samples (i.e., including untreated reference rabbits, hVEGF-challenged rabbits, anti-VEGF-treated hVEGF-challenged rabbits at both day 28 and day 56 time points). The PCA demonstrates distinct, non-overlapping expression patterns, with confidence of the samples analyzed being representative of labeled tissues from untreated reference rabbits that were harvested and tested. In addition, we investigated differentially expressed genes in the retina versus choroid tissue in these untreated reference rabbits. One outlier sample in the choroid tissue samples obtained from the untreated reference group was identified and excluded for further analysis (Supplementary Fig. S1). The expression of 29/35 target genes was significantly different (adjusted *P* < 0.001) in retina compared to choroid tissues derived from the untreated reference group (Fig. 4B). Expression of phosphatidylinositol-4,5-bisphosphate 3-kinase catalytic subunit alpha (*PIK3CA*), solute carrier family 16 member 14 (*SLC16A14*), and hypoxia-inducible factor 1-alpha (*HIF1A*) was higher in retina tissue compared to choroid tissue. Expression of 26 additional genes was higher in choroid tissue compared to the retina.

**Figure 4.**
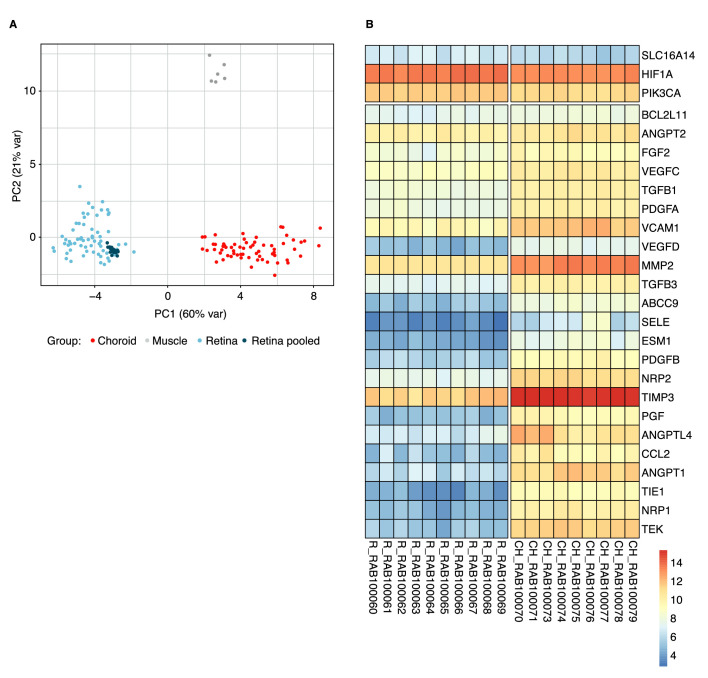
Gene expression analysis in retina and choroid tissue. PCA showing the distribution of choroid and retina from untreated reference rabbits, hVEGF-challenged rabbits, anti-VEGF treated hVEGF-challenged rabbits (all at both day 28 and day 56 time points), muscle control RNA, and retina pooled samples (A), and differentially expressed genes in retina versus choroid tissue of untreated reference rabbits (B). Significant regulation of genes (*P* < 0.001) in retina (C) and choroid (D) tissue after vascular leakage induction with hVEGF versus untreated reference rabbits can be seen. Genes are according to Reference Sequence (RefSeq) nomenclature.^37^

**Figure 4.**
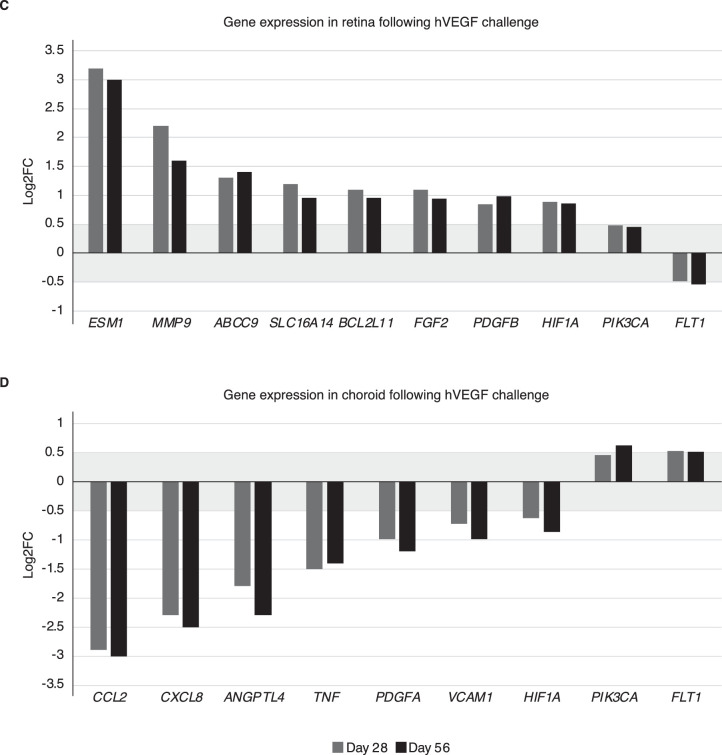
Continued.

Intravitreal injections of hVEGF significantly (*P* < 0.001) modulated 10 genes in retina (Fig. 4C) and nine genes in choroid tissue (Fig. 4D) versus untreated reference rabbit ocular tissue samples. Lower expression of *FLT1* (the gene encoding VEGFR-1) was discovered in both retina and choroid tissue following hVEGF challenge. The fold change was low (0.5); however, it was significant at both days 28 and 56. A similar trend was observed for *HIF1A* and *PIK3CA* in retina and choroid tissue at days 28 and 56. Figures 4C and 4D depict genes that were either upregulated in the retina or downregulated in the choroid from hVEGF-challenged rabbits at days 28 and 56 versus untreated reference rabbits.

### Gene Expression in Retina Tissue in hVEGF-Challenged Rabbits Receiving Anti-VEGF Treatments

**Figure 5.**
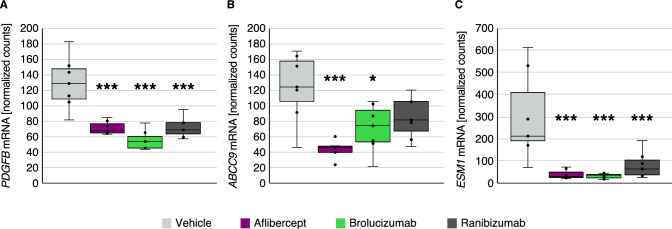
*PDGFB*, *ABCC9*, and *ESM1* mRNA expression in hVEGF-challenged rabbit retina tissue 28 days after anti-VEGF treatment. Vehicle-treated rabbit retina levels of (A) *PDGFB*, (B) *ABCC9*, and (C) *ESM1* mRNA compared to anti-VEGF-treated groups (*n* = 7 per group). *P < 0.05; ***P < 0.001. *ABCC9*, ATP binding cassette subfamily C member 9; *ESM1*, endothelial cell-specific molecule 1.

Further examination in NanoString analysis determined that mRNA expression of genes encoding ATP-binding cassette, sub-family C member 9 (*ABCC9*), Endothelial cell-specific molecule 1 (*ESM1*), and Platelet-derived growth factor subunit B (*PDGFB*) was lower in the retina of anti-VEGF–treated rabbits at day 28 following anti-VEGF treatment (Fig. 5) versus vehicle-treated rabbits. *ESM1* and *PDGFB* showed the greatest reductions following anti-VEGF treatment. In this analysis, only significant effects (adjusted *P* < 0.001) and median mRNA counts of more than 100 in vehicle-treated groups on day 28 and 56 were considered. In choroid tissue, there were no genes that showed significant differential expression at either day 28 or day 56 (Supplementary Table S1).

## Discussion

This study aimed to evaluate and profile gene and protein expression in the vitreous, retina tissue, and choroid tissue from a rabbit retinal vascular hyperpermeability model in which three anti-VEGF agents (ranibizumab, aflibercept, and brolucizumab) were administered prior to hVEGF challenge. The study also explored possible regulatory effects of anti-VEGF agents on selected target genes in the retina and choroid involved in angiogenesis, inflammation, vascular permeability and edema, as well as known factors involved in retinal disorders such as nAMD, DME, and RVO. The gene and protein analyses of ocular samples from this rabbit retinal vascular hyperpermeability model demonstrated that anti-VEGF therapies have effects beyond direct VEGF impact. The findings in this study, namely significant suppression of vitreous ANG2 protein and retina *ANGPT2* mRNA (Fig. 2), were consistent with leakage scoring results from Schubert et al.^35^ This study further confirms that, in high VEGF states in retinopathy, ANG2 levels increase, and, under suppressed VEGF concentrations, there is a corresponding decrease in ANG2 levels within the eye.

The results of this study support the hypothesis that ANG2 may be expressed secondary to primary insults to retinal vasculature by VEGF. In a study evaluating the potential influence of ANG2 on retinal endothelial cell tight junctional integrity, Regula et al.^23^ found that anti-ANG2 attenuated the steep reduction in transepithelial electrical resistance (TEER) observed following VEGF administration. Although the findings of Regula et al.^23^ supported a beneficial effect for additive anti-ANG2 with ANG1 in attenuating the adverse effects of VEGF on endothelial cell barrier function, there was no positive control of anti-VEGF as a comparison; hence, the study did not demonstrate whether additive anti-ANG2 provides any advantage above this threshold of activity. Moreover, the effect of ANG2 alone on TEER was not shown, which would have established the role of ANG2 in attenuating tight junctional integrity, thus confirming its contribution.^23^ Furthermore, in another study, although ANG2^−/−^ mice appear to exhibit protection from histamine/VEGF-induced permeability, ANG2 alone did not induce vascular permeability.^39^ This is in contrast to the well-known effects and role of VEGF (formerly known as vascular permeability factor^40^) on the induction of retinal and choroidal vessel permeability in retinopathies.

The ANG2/TIE2 pathway represents a secondary target for anti-angiogenic activity; however, it is currently debated whether targeting ANG2 in combination with VEGF could provide clinically meaningful benefits for patients with retinal vascular diseases.^28^^,^^32^ Clinical trials for nesvacumab were halted when this anti-ANG2 antibody, co-administered with aflibercept, failed to demonstrate superiority in visual acuity gains in both nAMD (ONYX^30^) and DME (RUBY^31^). Faricimab was developed as a bispecific agent that binds to both VEGF and ANG2.^23^ Phase 3 clinical trials for patients with nAMD (TENAYA and LUCERNE, T&L^34^) and DME (YOSEMITE and RHINE^41^) treated with faricimab at variable treatment intervals demonstrated non-inferiority to aflibercept. Comparable efficacy of faricimab with ranibizumab was demonstrated in a phase 2 trial of patients with nAMD (STAIRWAY^42^); however, the superiority of faricimab over ranibizumab was not demonstrated in a different phase 2 trial of patients with nAMD (AVENUE^43^). The superiority of faricimab to ranibizumab (with regard to visual acuity gains) was demonstrated in patients with DME (BOULEVARD^44^).

Although up to 80% of patients were reported to receive faricimab at intervals of every 12 weeks in T&L, this may not reflect the proposed additional benefit of targeting ANG2. First, in T&L, patients were, on average, representative of an easier-to-treat population, having marginally higher baseline vision, thinner retinas, and smaller choroidal neovascularization lesions than typically observed in clinical trials.^34^^,^^45^ Second, these effects could be attributed to the higher molar doses of ranibizumab, given that the anti-VEGF arm of faricimab is ranibizumab.^23^ The calculation of the estimated contribution of ranibizumab to the 6-mg clinical dose of faricimab, by taking into account the relative molecular weight contributions per entity, proceeds as follows: (48 kD ÷ 150 kD) × 6 mg = 1.92 mg. This results in an approximated 2-mg dose of ranibizumab, which is ∼fourfold the clinical dose of ranibizumab (0.5 mg). The HARBOR study evaluated the effectiveness of ranibizumab 2 mg versus ranibizumab 0.5 mg in patients with nAMD over 24 months.^46^ Consistent with the faricimab phase 2 trials,^42^^,^^43^ visual acuity gains (measured by Early Treatment of Diabetic Retinopathy Study letters) of the ranibizumab 2 mg pro re nata (PRN) group were nearly identical to the ranibizumab 0.5 mg PRN group (+7.6 letters vs. +7.9 letters, respectively, at 24 months) and were accompanied with a reduction in number of injections when compared to the 0.5-mg dose (11.2 injections vs. 13.3 injections, respectively, at 24 months).^46^ In summary, perceived durability advantages reported with faricimab could potentially be accounted for by the larger dose of anti-VEGF in the molecule, as well as the presence of an easier-to-treat population in T&L due to baseline characteristics.

Given that the present study has shown that aflibercept, ranibizumab, and brolucizumab significantly suppress ANG2 levels (which were elevated in the retinal vascular leakage model), and in light of the non-inferiority of faricimab to aflibercept and ranibizumab in clinical trials, there is a lack of convincing evidence that suppression of ANG2 confers any further clinical benefit. Recently, health authorities such as the FDA have stated that the clinical relevance of suppression of ANG2 over the known and confirmed activity of VEGF suppression has not been shown.^32^ In addition (to our knowledge), there are no anti-ANG2 monotherapies approved for the treatment of retinal diseases.

Interestingly, FGF2 was significantly suppressed in vitreous solely by aflibercept, which also resulted in the greatest suppression of *FGF2* mRNA in the retina versus brolucizumab and ranibizumab on day 28 (Fig. 3). FGF2 has been reported to be a contributor to pathologic fibrosis of the retina, and preclinical studies have explored its potential as a therapeutic target in rodent retinopathy models.^47^ Fibrosis and atrophy are damaging processes that are observed in the progression of AMD and diabetic eye diseases.^48^ These results provide insights into the multiplicity of relevant retinopathy mechanisms that are potentially addressed with aflibercept.

In addition to ANG2, Figure 5 describes an exploratory analysis of several different genes modulated in the rabbit model of vascular hyperpermeability following anti-VEGF administration. *PDGFB* was significantly downregulated in retina tissue in this study. Elevated platelet-derived growth factor subunit B (PDGFB) and activated forms of the PDGF receptor have been observed in the plasma of patients with nAMD (Boyer et al., *IOVS*. 2009;50:ARVO E-abstract 1260),^49^ suggesting that PDGFB could be involved in the pathogenesis of nAMD. CAPELLA^50^ and FOVISTA^51^ were recent clinical trials examining dual VEGF and either PDGFB receptor (CAPELLA) or PDGFB (FOVISTA) inhibition, which were developed based on the principle of dual targeting to provide additional benefit over targeting VEGF only. Although initial phase 2b combination therapy results were promising,^52^ both the FOVISTA phase 3 and the CAPELLA phase 2 trials failed to meet their primary endpoints of superior improvements in visual acuity in patients with nAMD compared to patients receiving ranibizumab alone or aflibercept alone, respectively. Nonetheless, *PDGFB* upregulation is likely to be a secondary factor that is robustly suppressed by anti-VEGF therapies.

In this study, *ABCC9* expression was increased following VEGF-induced vascular leakage and suppressed following the administration of anti-VEGF agents. *ABCC9* has been identified to contribute to retinal neovascularization in a microarray analysis of two murine oxygen-induced retinopathy models,^53^ and was determined to be downregulated following aflibercept administration in the mouse oxygen-induced retinopathy model in the study by Rojo Arias and Jaszai.^2^ *ABCC9* encodes subunits of adenosine triphosphate (ATP)-sensitive potassium channels and may confer enhanced retinal resistance against severe ischemic injury^54^; hence, downregulation may be a consequence of improved retinal oxygenation, thus removing the demand for ABCC9 activity.

Similarly, *ESM1* was suppressed in the present study following anti-VEGF agent administration. *ESM1* was observed to be overexpressed in a mouse model of oxygen-induced retinopathy^55^ and was downregulated following aflibercept administration in the study by Rojo Arias and Jaszai.^2^ Increased expression of *ESM1* was implicated in increased proliferation and tumorigenesis in an in vitro study of an epithelial human breast cancer cell line^56^; thus, the downregulation of *ESM1* by anti-VEGF agents may support the regeneration of the retinal microvascular network.^2^

Although the rabbit retinal vascular hyperpermeability model used in this study is reflective of the clinical situation in part, such that elevated, dysregulated VEGF incites vascular permeability, edema, and neovascularization, and ANG2 does not trigger an angiogenic or vascular permeability response alone,^57^ the model does present with some limitations. Unlike the clinical situation, in which the retinal disorder is present prior to administration of the anti-VEGF therapy, healthy rabbits receive an initial anti-VEGF treatment at baseline of the study, several days prior to induction of vascular leakage with the administration of VEGF. Although the model does not address the chronic pathology of retinal disorders, it provides valuable information regarding phenotypical aspects of retinal disorders, such as the consequences of pathologic retinal vascular leakage. To limit confounders such as potential differential cross-reactivity for the different anti-VEGF agents in rabbits, hVEGF was used for leakage induction, thus mitigating this weakness. Potential cross-reactivity differences were further mitigated due to the high sequence of homology of the various targets of the study (including ANG2 and FGF2) between rabbits and humans, which facilitated detection of these agents in the rabbit ocular tissues. The methodology was modified and optimized to address any apparent differences in assay recovery between humans and rabbits.

Consistent with the in vivo results showing that aflibercept provided the longest suppression of vascular leakage,^35^ aflibercept in general also exhibited the most durable and robust suppression of *VEGF* and *ANG2*, as well as the suppression of other relevant target genes within the retina/choroid. Future studies could look to explore the involvement of microRNA-351 in anti-VEGF signaling, given that the common targets of this microRNA include both *VEGF* and *ANG2*. Crosstalk among these three elements has been found to play a role in hypoxia-induced microvascular response in an in vivo study using a chorioretinal endothelial cell line.^58^ Additionally, it would be of interest to assess the ANG2 suppression profile at day 42, when aflibercept continued to show significantly greater suppression of vascular leakage than brolucizumab and ranibizumab to further support association.^35^

In summary, the centrality of the VEGFR pathway in the efficacy of anti-VEGF treatment in retinal disorders was reinforced in this study. Various growth factors and target genes known to modulate different retinal disorder phenotypes were shown to be increased secondary to VEGF-induced vascular leakage in the rabbit model. The levels of these upregulated growth factors and target genes were suppressed by anti-VEGF treatment, suggesting that direct inhibition of these secondary targets may not be clinically relevant (for example, the suppression of PDGFB and ANG2) in the vitreous, retina, and choroid, as this is currently provided by anti-VEGF treatment alone. Indeed, none of the anti-VEGF agents in this study directly bound ANG2, yet all suppressed intraocular ANG2 protein and retinal/choroidal gene expression, with aflibercept demonstrating the most robust suppression.

## Supplementary Material

Supplement 1

Supplement 2
