# Supplementary material for: Aflibercept Suppression of Angiopoietin-2 in a Rabbit Retinal Vascular Hyperpermeability Model
Source: Transl Vis Sci Technol. 2023 May 16;12(5):17. doi: 10.1167/tvst.12.5.17 (PMC10198290; doi:10.1167/tvst.12.5.17)
Supplement: Supplement 1 [file tvst-12-5-17_s001.docx]

**Supplementary Fig. S1.** Exclusion of outlier rabbit from choroid tissue samples obtained from untreated reference group.


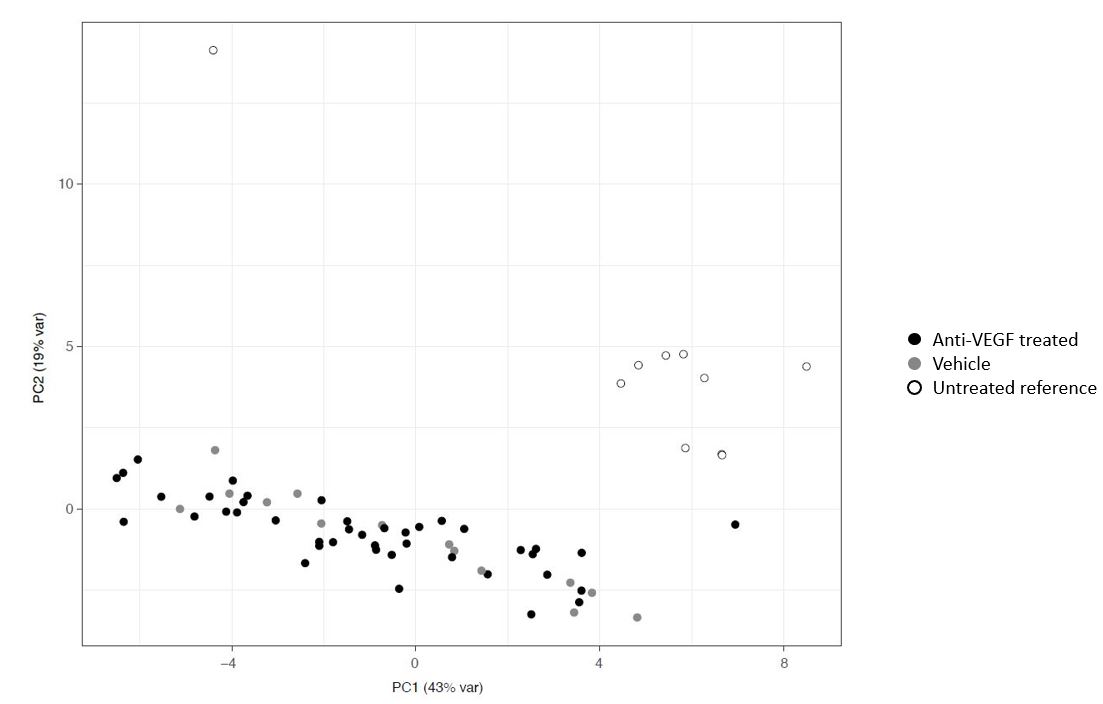


Principal component analysis showing distribution similarity in expression of the 35 target genes in choroid tissue between samples from each arm of the study. The sample from the untreated reference group on the top left is an outlier when compared with all other untreated reference samples (by two standard deviations).

VEGF, vascular endothelial growth factor.

**Supplementary Fig. S2.** *ANGPT2* and *FGF2* mRNA expression in hVEGF-challenged rabbit choroid tissue 28 days after anti-VEGF treatment.


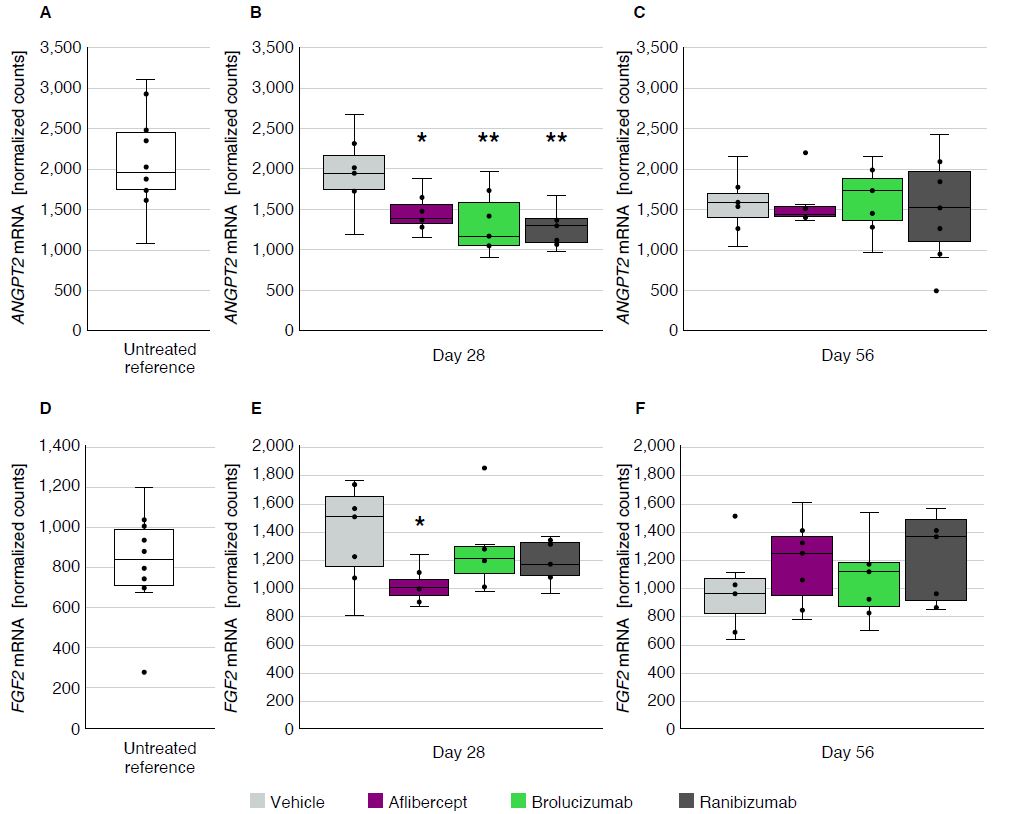


*ANGPT2* and *FGF2* gene expression was determined by mRNA analysis (normalized counts) in choroid (**A**) tissues derived from untreated reference rabbits (*n* = 10), and after VEGF challenge and vehicle or anti-VEGF treatment on Day 28 (**B**) and Day 56 (**C**) in the retina (*n* = 7 per group). **P* < 0.05; ***P* < 0.01.
*ANGPT2*, angiopoietin-2; *FGF2*, basic fibroblast growth factor; (h)VEGF, (human) vascular endothelial growth factor.
